# Effectiveness of Silver Diamine Fluoride for Early Childhood Caries Among Children Aged 24 to 72 Months: Protocol for a Randomized Controlled Trial

**DOI:** 10.2196/46144

**Published:** 2023-06-07

**Authors:** Amitha Basheer N, Praveen S Jodalli, Namratha Nayak, Aparna K S, Avinash R Badekkila

**Affiliations:** 1 Department of Public Health Dentistry Yenepoya Dental College Yenepoya (Deemed to be University) Mangalore India; 2 Department of Public Health Dentistry Manipal College of Dental Sciences Mangalore Manipal Academy of Higher Education Manipal India; 3 Department of Periodontology Manipal College of Dental Sciences Manipal Manipal Academy of Higher Education Manipal India

**Keywords:** silver diamine fluoride, children, caries, childhood, dental, dentist, oral health, child, pediatric, RCT, randomized, fluoride, prevention

## Abstract

**Background:**

Dental caries is a serious public health issue globally. It is one of the most prevalent chronic disease in children worldwide. An important public health concern is the existence of one or more decayed, missing, or filled tooth surfaces in any primary teeth of preschool children. Early childhood caries (ECC) can be halted with the use of silver diamine fluoride (SDF) solution. Previous research has indicated that it may have a preventive impact in the treatment of ECC. It is well known that 38% silver diamine fluoride (SDF) is helpful at preventing dental caries. On the other hand, there is not enough evidence on SDF’s ability to prevent caries in primary teeth. To date, no well-planned clinical study has been carried out to investigate SDF’s impact on caries protection.

**Objective:**

The study’s objective is to evaluate and compare the efficacy of 12%, 30%, and 38% silver diamine fluoride in preventing ECC in children aged 24 to 72 months in Mangaluru Taluk.

**Methods:**

This is a single-center, randomized, active-controlled, parallel-group pragmatic trial. Children attending preschool in Mangalore Taluk aged 24 to 72 months will be included in the study. There will be three study groups: group 1 will receive 12% SDF semiannually; group 2 will receive 30% SDF semiannually; and group 3 will receive 38% SDF semiannually. After 6 and 12 months, the principal examiner will conduct a clinical examination of the teeth with a visual and tactile assessment. The effectiveness of the various concentrations of SDF will be determined after 12 months.

**Results:**

The research was funded in September 2020, and data collection started in September 2022. As of February 2023, 150 participants have enrolled in the study. The project is still underway, and it is expected to be completed in December 2023.

**Conclusions:**

Uncertainty surrounds the efficacy of 38% SDF in preventing ECC. Consensus-Based Clinical Case Reporting (CARE) guidelines recommending the use of SDF for ECC prevention will be modified if the findings are as expected. Additionally, since the findings will be broadly disseminated, more nations will implement the use of SDF, easing the burden of ECC on the entire world. Future research on the treatment and prevention of ECC will benefit from the study results. If SDF is successful in preventing caries in a classroom or community context, it will mark an important turning point for preventive dentistry.

**Trial Registration:**

Clinical Trial Registry of India CTRI/2020/02/023420; https://tinyurl.com/3ju2apab

**International Registered Report Identifier (IRRID):**

PRR1-10.2196/46144

## Introduction

### Background

Dental caries is a complex, bacteria-driven, chronic, site-specific, and significant process brought on by a physiological imbalance between minerals in teeth and the fluid in plaque [1]. It is a progressive condition that results from the demineralization of dental enamel caused by the acids generated during the metabolism of sugars by cariogenic bacteria [2] and is one of the most prevalent chronic childhood illnesses; in developing nations, many decayed teeth go untreated. The World Health Organization (WHO) estimates that there are an average of 2.4 decaying, missing, and filled teeth per person worldwide. Dental caries continues to be a significant problem in the majority of developed nations, impacting 60% to 90% of school-aged children. In many industrialized nations, there have been encouraging trends in the fight against pediatric dental caries. In India, where more than 70% of the population lives in rural areas with few health services and restorative therapy is expensive, parents tend to neglect their children’s dental needs, leading to untreated dental caries [1].

The term early childhood caries (ECC) has been used to describe the presence of one or more decayed, restored, or missing primary teeth in a child younger than 6 years [3]. The American Dental Association has classified the presence of one or more decayed, missing, or filled tooth surfaces in any primary tooth in a preschool-aged child as a major public health concern. If ECC is not addressed, it may affect a person’s quality of life in terms of dental health, as well as their body weight, growth, attendance at school, and academic achievement. Measures to prevent and treat severe ECC are essential for both general and dental health because it is a major indicator of adult caries [4].

Poor oral health–related quality of life, low BMI, bad scholastic results, and high absenteeism from school are just a few of the harmful impacts of untreated dental caries that can have an impact on physical development. Due to their vulnerability and reliance on their caretakers for both nutritional requirements and mouth cleanliness, children are most at risk of developing dental caries. Dental caries frequently exhibits no symptoms in its early phases, but if left unchecked, it can cause excruciating pain and potentially fatal infections [3].

ECC prevalence varies from population to population. The illness is most prevalent and severe in East Asia, where prevalence rates in children aged 3 years range from 36% to 85%. In India, 44% of children aged between 8 and 48 months have ECC. Despite tremendous advancements in the field of caries control in recent decades, a high prevalence of ECC in young infants has been observed. It is thus a major social concern; disease control can be accomplished if the demographic groups with the greatest needs are identified. The prevalence of ECC overall in India is 49.6% according to an analysis of all included studies. While in Karnataka state, the prevalence of ECC was reported to be 48.79% [5].

The current cost of treating oral diseases, such as dental caries, places a heavy financial burden on both households and health care providers. Additionally, because of the high expense of dental treatment, elderly adults, socially excluded groups, and children living in poverty are more likely to develop oral diseases. Moreover, even if people have access to therapy, the current course of action for children with severe caries includes giving them local anesthesia to ease their pain and worry. Such treatments have been linked to poor oral health, dental anxiety, and a higher risk of dental caries in children’s permanent teeth in later life. In order to prevent and manage caries and encourage health and well-being, new, accessible, and equitable methods are required [6].

Nonrestorative intervention is a potential alternative method for treating caries in primary molars. Silver diamine fluoride (SDF) has been used successfully to stop the progression of carious lesions in people who cannot tolerate invasive treatment, including children, the elderly, people with medical conditions, and people who require additional care and support. Even though the mechanism of action of SDF is debatable, SDF treatment has been shown to support tooth desensitization and to arrest carious lesions through dentinal tubule blockage, cariogenic bacterial death, remineralization of the demineralized tooth, and inhibition of dentinal collagen degradation. Hence, SDF is a cost-effective, reliable, secure, and efficient caries-prevention substance [6].

Japan authorized the use of silver diamine fluoride (SDF) as a medication in the 1960s. It has been used for many years to cure dental caries in China, Australia, Brazil, Argentina, and Australia. The US Food and Drug Administration authorized the first SDF product for use in the country in 2014. Since 1969, SDF has been used to prevent tooth decay in young children’s primary teeth and pit and fissure caries in developing permanent molars, as well as to treat root caries in geriatric patients. In addition to treating caries, SDF is also used to sterilize infected root canals and treat dental hypersensitivity. It can be applied directly to a carious lesion to arrest the spread of caries or to a caries-prone surface to prevent caries [7].

Although some drawbacks of SDF have been reported, such as staining, its safety has been strongly backed by the fact that not a single untoward event has been connected to its use since it was introduced in Japan. Recent Hong Kong–based randomized controlled studies showed that SDF can decrease carious lesions among children, if SDF is administered twice a year at a concentration of 38%. This method is advised because it can arrest caries without leading to excessive staining. However, after 6 months, it was discovered that a single application of SDF at a concentration of 38% was effective in halting dentinal caries in the primary anterior teeth of preschoolers [6].

SDF has the ability to prevent discomfort, sepsis, and the requirement for extractions if applied early, when the cavity is evident but has not yet resulted in permanent pulpal injury. A 38% SDF solution is more efficient than a solution with a lower concentration, and biannual application is superior to annual application. The use of SDF encourages participation and community support from parents who do not seek dental consultation for their children. Additionally, silver-modified atraumatic restorative therapy is an appealing option that improves treatment results. SDF can increase accessibility and equity in dental care, particularly for people from low socioeconomic backgrounds [6].

A study done by Fung and colleagues in 2018 [8] compared 12% and 38% SDF and found that decay was more substantially arrested when using the higher concentration. Neither that report nor the clinical trial registry listing provides the details of product origin. Further, the investigators did not independently verify the concentrations, making it impossible to generalize from their findings. More research is required to completely evaluate the efficacy of SDF and identify the most effective concentration in order to set guidelines. This study will add on to existing literature, as the SDF product used in our study has not been trialed before. The procedure can be used in basic care settings and could ease the population’s burden of untreated tooth decay. SDF has the potential to play a significant role in comprehensive and successful preventative initiatives to achieve the UN Sustainable Development Goals. We hypothesize that both 12% and 30% SDF will have comparable efficacy with 38% SDF.

### Objective

To assess and compare the effectiveness of 12% SDF, 30% SDF, and 38% SDF for treatment of early childhood caries among children aged 24 to 72 months in Mangaluru Taluk.

## Methods

### Trial Design and Protocol Development

**Figure 1:**
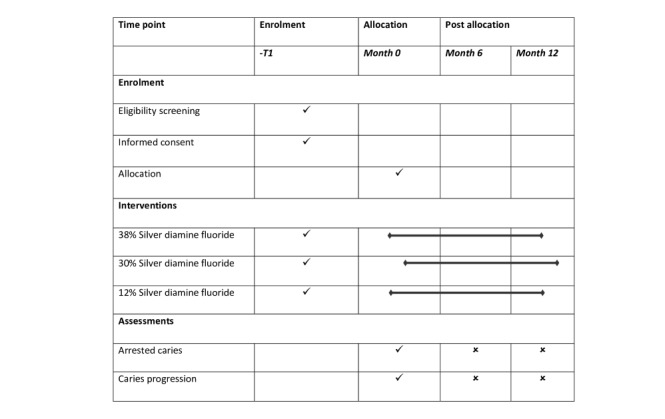
Schedule of enrollment, intervention, and assessment of groups that received 38%, 30%, and 12% silver diamine fluoride.

This is a single-center, randomized, active-controlled, parallel-group pragmatic trial. This clinical trial protocol’s design and report adhere to the SPIRIT (Standard Protocol Items: Recommendations for Interventional Trials) declaration. The trial schedule is shown in Figure 1.

### Study Setting

The study is being carried out at preschools of Mangaluru Taluk, South India. The dental examinations are performed after receiving confirmation of participation from preschool teachers or caregivers, and informed consent forms (Multimedia Appendix 1) are sent to the parents of the eligible children. Signed parental consent is collected before the intervention starts.

### Participant Selection

Children attending preschools in Mangalore Taluk aged from 24 to 72 months are included. Additional inclusion criteria are as follows: at least one soft caries lesion (ie, cavitated active caries lesion) that is small (less than half of the mesial or distal or less than one-third of the buccal, lingual, or occlusal surfaces). The exclusion criteria are pulpal exposure, history of pain, nonvital teeth, swelling or fistula, silver allergy, arrested caries or inactive lesions, teeth at the period of shedding, restored teeth, secondary caries, fluorosis, enamel hypoplasia, uncooperative behavior, physical or mental disability, and a lack of signed informed consent.

### Baseline and Follow-up Oral Examinations

Before conducting the trial, the principal investigator was trained and calibrated by experienced dental public health specialists in the Caries Assessment Spectrum and Treatment (CAST) index. Training and calibration sessions included theoretical aspects and were followed by clinical examination of a group of 10 subjects from the outpatient department. The subjects were reexamined by the principal investigator and the guide (an experienced dental public health specialist) after 1 week. The κ coefficient values for intraexaminer and interexaminer reliability were 0.88 and 0.80, respectively, reflecting a high degree of conformity in the observations.

The examinations in this study are performed by a single examiner with the help of a recording device in preschools in Mangaluru Taluk. The study sample comprises 3 groups of children aged 24 to 72 months. After baseline examination, eligible children are randomly allocated to 1 of the 3 groups using the lottery method. Group 1 receives 12% SDF semiannually, group 2 receives 30% SDF semiannually, and group 3 receives 38% SDF semiannually. Dental caries will be measured using the CAST index [9].

A CAST score of 0 is given to sound teeth (ie, with no visible evidence of a distinct carious lesion); a score of 1 for pits or fissures that are at least partially covered with a sealant material; a score of 2 for cavities that have been restored with a direct or indirect restorative material; a score of 3 for distinct visual change in the enamel only or clearly visible caries-related discoloration, with or without localized enamel breakdown; a score of 4 for internal caries-related discoloration in the dentine, with the discolored dentine visible through the enamel, which may or may not exhibit a visible localized breakdown; a score of 5 for distinct cavitation into the dentine with the pulp chamber showing intact pulp; a score of 6 for involvement of the pulp chamber with distinct cavitation reaching the pulp chamber or with the presence of only root fragments and abscess or fistula; a score of 7 for pus-containing swelling or a pus-releasing sinus tract related to a tooth with pulpal involvement; a score of 8 for teeth that have been removed because of dental caries; and a score of 9 for teeth that do not match any of the above descriptions.

All tooth surfaces, including the buccal, lingual, mesial, distal, and occlusal surfaces for posterior teeth, are evaluated for caries. Miller’s criterion is used to assess whether active caries are present; Miller’s criterion suggests that a carious lesion is termed active if a blunt University of North Carolina–15 probe can easily enter it; a carious lesion is defined as halted when the dentine cannot be penetrated.

The size of the lesion is categorized as small or large at the tooth-surface level (less than one-third of the buccal, lingual, or occlusal surface, or less than half of the mesial or distal surface).

### Intervention

#### Drug Description

The SDF in this study is Caries Stop (Biodinamica); it was used at product concentrations of 12%, 30%, and 38%. The investigators did not independently verify the concentrations stated by the manufacturer. The product is composed of fluoridric acid, silver nitrate, ammonia hydroxide and deionized water.

#### Application of SDF

Primary teeth that have cavities are separated and kept dry using cotton rollers. A plastic dish is prepared that contains 1 drop of SDF. Using a tiny brush, the SDF is administered to the carious lesion for 2 minutes. The excess is eliminated by gently wiping with another cotton pellet. The children are requested to avoid any dietary intake for an hour after application. At 6 months after the first application, a second application will be carried out. After 6 and 12 months, the principal examiner conducts a clinical examination of the teeth with a visual and tactile assessment. After a year, the efficacy of the various SDF concentrations will be evaluated.

### Outcome Measure

The primary outcome measure is caries arrest at the twelfth month of application of SDF. The secondary outcome measure is the caries increment (ie, CAST index).

### Effect Modification

A parental questionnaire is administered at baseline regarding the children’s oral hygiene habits (eg, tooth brushing), fluoride agent use (eg, fluoride toothpaste), dietary habits (eg, bottle feeding), and source of drinking water.

### Randomization and Treatment Allocation

**Figure 2:**
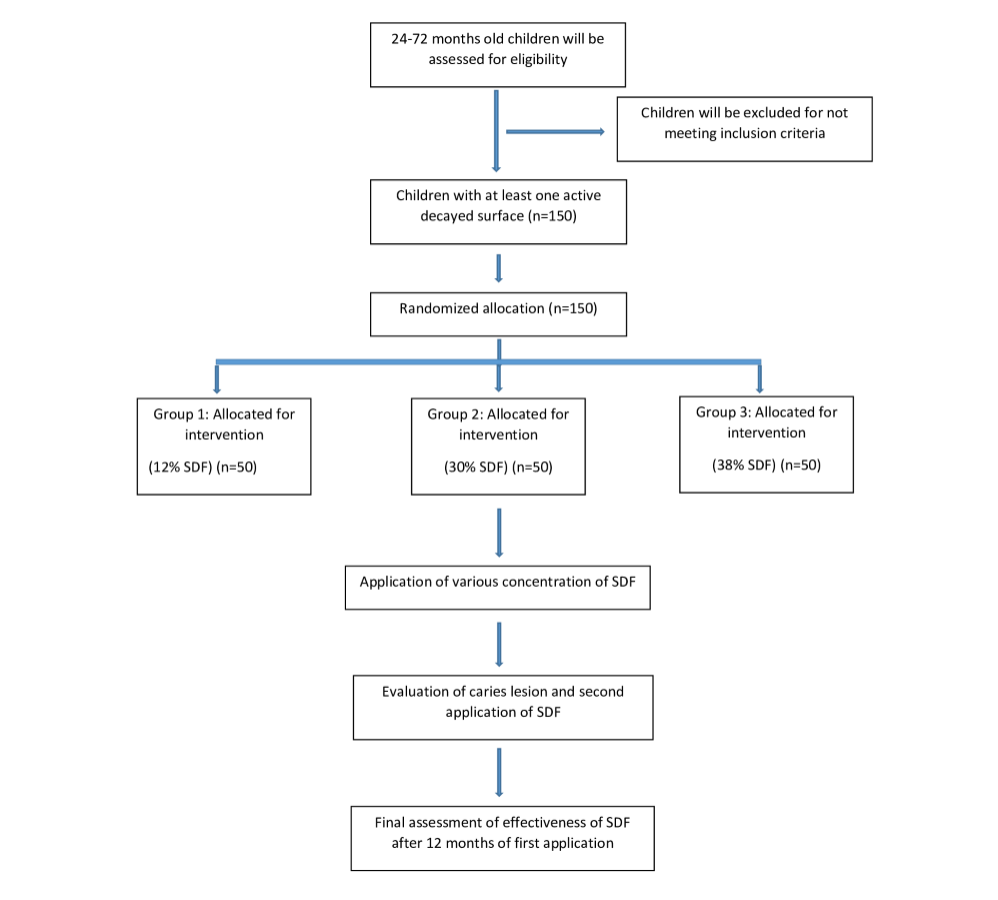
Enrollment and allocation of sample. SDF: silver diamine fluoride.

The study is being conducted in preschools in Mangaluru Taluk. Multistage random sampling was used to select the preschools, with the following stages: (1) Mangaluru Taluk was divided into north and south zones; (2) preschools in Mangaluru Taluk were divided into government schools, government-aided private schools, and non–government-aided private schools; and (3) children were selected randomly from 437 preschools (n=218 from the south zone and n=219 from the north zone). Children were randomly allocated to 1 of the 3 groups using the lottery method. The study sample comprises 150 students aged from 24 to 72 months attending preschools in Mangaluru Taluk, including 50 students in group 1 (12% SDF semiannually), 50 students in group 2 (30% SDF semiannually), and 50 students in group 3 (38% SDF semiannually), as shown in Figure 2.

### Random Allocation, Concealment, Masking, and Blinding

Allocation concealment was done using the sequentially numbered opaque sealed envelope method. A random code for each participant was put in an opaque package as part of a randomization system. Masking is used for the products, which are indistinguishable in terms of consistency, packaging, and labeling. The researchers applying the products are unaware of what product is being applied. A dentist’s aide opens the envelope, and the materials are then prepared after the child’s designated actions in accordance with their group allocation. The children and parents are blinded regarding their grouping. The principal examiner, who is blinded to the participating children’s group assignment, performs a clinical evaluation of the children at baseline and at semiannual follow-up exams. The dentist’s aide is unblinded to the interventions and aids in allocating the participants to the principal examiner. The participant’s allocated intervention during the trial will be revealed to the principal examiner only after the follow-up is completed.

### Sample Size Estimation

Based on the previous studies, the anticipated proportion of sound occlusal surfaces at baseline that develop into cavitated dentin lesions at the 30-month follow-up is approximately 25% [3].

Sample size calculation was performed with G* Power 3 (Heinrich-Heine-Universität Düsseldorf). Based on a 5% level of significance, power of 80%, effect size of 0.63, and 3 groups, the required sample size is 120, including 40 in each group. Considering a drop-out rate of 20%, the final sample size is 150, as shown by the following equation: final sample size = ND = N / (1 – d), where *N* is the sample size before drop-out, *d* is the expected drop-out rate, and *ND* is the sample size considering drop-out. Thus, ND = 120 / (1 – 0.2) = 120 / 0.8 = 150, and the total sample required for intervention is 150 (50 in each group). The allocation ratio is 1:1:1. The participants are allocated to the 3 groups equally.

### Statistical Analysis

Data analysis will be done using SPSS (version 23.0; IBM Corp). Continuous variables will be presented as the mean (SD). Categorical variables will be presented as frequencies and percentages. To confirm normality, the Shapiro-Wilk and Kolmogorov-Smirnov tests will be used. A one-way analysis of variance (ANOVA) statistical test will be used to compare the means of continuous outcome variables if the data follow a normal distribution. Nonparametric tests (ie, the Kruskal-Wallis statistical test) will be used in the data analysis in cases when there is skewed distribution of data. Post hoc tests will be performed with an ANOVA or the Kruskal-Wallis test.

Binary logistic regression will be performed. The dependent variable will be whether the lesion is arrested or not at the 12-month examination, and the independent variables will include the children’s oral hygiene habits (eg, tooth brushing), fluoride agent use (eg, fluoride toothpaste), dietary habits (eg, bottle feeding), and source of drinking water.

### Ethical Considerations

Ethical approval has been obtained from the Institutional Review Board (Ethics Committee II) of Yenepoya (Deemed to be University) for protocol version 3 (YEC2/175). Research aides will serve as contact people and be involved in all phases of the trial. Parents or legal caretakers of the children are given an information sheet outlining the purpose of the study, the specifics of the research, and the processes after the principals consent to participate in the study. Parents are informed of the side effects of the treatment, notably staining of the tooth. Their queries regarding the side effects are addressed. Before beginning this trial, informed consent is obtained. The participants, including the children and their guardians, are free to withdraw at any time during the study. They are also allowed to pursue additional dental care according to their own decisions and financial means.

Caries assessment is done with aseptic precautions and without any harm to the patient. The application of 12% SDF, 30% SDF, or 38% SDF is also done with aseptic precautions and without any harm to the patient. If any complications take place, the child is treated free of charge by the principal investigator.

The study data will be deidentified for privacy and confidentiality protection. No compensation will be offered to the study participants.

The drugs were sponsored by Biodinamica. This drug company has no role in the research.

The Drug Controller General of India does not normally restrict the use of topically applied drugs. Currently, there are also no official guidelines announced for SDF use by dental professional associations or research bodies [10].

### Risks of the Study

There are no major known risks to the study. Minor risks, such as black stains on the carious tooth surface or temporary stains on the skin, will occur. Temporary stains on the skin disappear within 48 hours without treatment.

### Withdrawal Criteria

During semiannual applications, clinical examination for caries will be done. If any pulpal involvement or abscess is noticed, appropriate treatment will be provided by the principal investigator. The children will be given the right to refuse or withdraw at any point in time.

### Maximum Dose and Safety Margin

The margin of safety for dosing is of paramount concern. The typical lethal dose, 50% (LD 50), for oral administration is 520 mg/kg. One drop (25 µL) contains 9.5 mg SDF and is sufficient to treat 5 teeth. The dose will be 0.95 mg/kg, and assuming that the smallest children with caries weigh between 10 kg and 15 kg, the relative safety margin for administering a whole drop to a child weighing 10 kg is therefore 380 mg/kg LD 50 / 0.95 mg/kg dosage, for a 400-fold safety margin. The actual dose will probably be much lower. One drop (25 µL) per 10 kg per treatment session is the suggested upper limit. The largest applied dose for 3 teeth is 2.37 mg, allowing for more than 400 applications [11].

### Adverse Effects

Information on adverse effects of SDF treatment—including tooth or gum pain, gum swelling, gum bleaching (ie, becoming whiter), and systemic toxicity (nausea, vomiting, and generalized discomfort)—is collected through a parental questionnaire within a week of SDF or placebo application at baseline and at 6- and 12-month follow-ups. If there is an acute systemic illness associated with SDF treatment, the parents are asked to immediately contact the principal investigator by telephone. Oral examinations will also be performed at baseline and at 6- and 12-month follow-ups. An examiner will evaluate the hard and soft tissues to establish changes and visible alterations during the clinical examination. All changes will be recorded on a case report form.

## Results

The research was funded in September 2020, and data collection started in September 2022. As of February 2023, 150 participants were enrolled in the study. The project is still underway and is expected to be completed in December 2023. The findings of this study will be beneficial for future studies and the management of early childhood caries.

The information obtained from the study will be submitted to the institution and to the concerned authorities. The details of the study will be published in journals and presented at conferences.

## Discussion

### Principal Aims

The purpose of this single-center, randomized, active-controlled, 3-arm pragmatic trial is to evaluate and compare the efficacy of 12%, 30%, and 38% SDF in preventing early childhood caries in children aged 24 to 72 months. We are using 38% SDF, which is thought of as the standard of care for preventing ECC, in a positive control group to determine if 30% and 12% SDF are superior.

Our hypothesis is that 38% SDF will have a superior effect compared to 30% and 12% SDF in stopping ECC, because it has a fluoride concentration that is much higher (44,800 ppm) than 30% (35,400 ppm) or 12% (14,100 ppm) SDF. The standard of care will be altered if the results are as expected. Because every child who enrolls in the study should have the right to obtain an efficient preventative strategy, there is no negative control group.

In this study, we evaluate the ability of 12%, 30%, and 38% SDF to arrest ECC. The use of SDF solutions in a clinical setting has been deemed safe, and the application process is noninvasive. Despite the fact that the participants in this study are recruited at a young age, few ethical questions have been raised.

In order to examine the efficiency of 12% and 38% SDF in halting dentin caries in the primary teeth of preschool children, Fung et al [8] undertook a randomized clinical experiment. Other recent research indicates that 38% SDF is more effective than 12% SDF. In a clinical trial, Vollú et al [12] compared the effectiveness of 30% SDF and atraumatic restorative treatment in halting dentine caries in the primary molars of preschoolers. Recent research revealed that 30% SDF could stop caries lesions with effects comparable to those of atraumatic restorative treatment.

The findings of this trial, performed in young children who are in good health, may not be generalizable to other populations of children, such as those who require special medical attention or are older, which is a limitation of this study. It will be necessary to perform additional research on the preventive impact of SDF in individuals with special health care requirements. The generalizability of the study will also be limited by the fact that clinical trials in different countries use different parameters for caries assessment.

The study outcomes will be widely disseminated, which should encourage the adoption of SDF in other nations and lessen the global burden of ECC. SDF may even become a crucial component of comprehensive and successful preventative programs to achieve the UN sustainable development goals.

### Conclusions

The findings of this study will be beneficial for future studies on the management and prevention of ECC. If SDF is found to be effective in a school or community setting for caries prevention, it will be a significant milestone for preventive dentistry.

## Appendix

Multimedia Appendix 1 Informed consent form given to parents.

## Abbreviations

ANOVA: analysis of variance
CARE: Consensus-Based Clinical Case Reporting Guideline
CAST: Caries Assessment Spectrum and Treatment
ECC: early childhood caries
SDF: silver diamine fluoride
WHO: World Health Organization

## Data Availability

The data sets generated during and/or analyzed during this study are available from the corresponding author on reasonable request.
