# Correction: Effectiveness of Silver Diamine Fluoride for Early Childhood Caries Among Children Aged 24 to 72 Months: Protocol for a Randomized Controlled Trial

**DOI:** 10.2196/50744

**Published:** 2023-07-26

**Authors:** Amitha Basheer N, Praveen S Jodalli, Namratha Nayak, Aparna K S, Avinash R Badekkila

**Affiliations:** 1 Department of Public Health Dentistry Yenepoya Dental College Yenepoya (Deemed to be University) Mangalore India; 2 Department of Public Health Dentistry Manipal College of Dental Sciences Mangalore Manipal Academy of Higher Education Manipal India; 3 Department of Periodontology Manipal College of Dental Sciences Manipal Manipal Academy of Higher Education Manipal India

In “Effectiveness of Silver Diamine Fluoride for Early Childhood Caries Among Children Aged 24 to 72 Months: Protocol for a Randomized Controlled Trial” (JMIR Res Protoc 2023;12:e46144) the authors noted two errors.

1.) In the originally published manuscript, affiliation 2 appeared as follows:

Department of Public Health Dentistry, Manipal College of Dental Sciences Mangalore, Manipal Academy of Higher Education, Mangalore, India

This has been corrected to:

Department of Public Health Dentistry, Manipal College of Dental Sciences Mangalore, Manipal Academy of Higher Education, Manipal, India

2.) In the originally published manuscript, the postal code for the Corresponding Author address appeared as follows:

Mangalore, 575001

This has been corrected to:

Manipal, 5756104

The correction will appear in the online version of the paper on the JMIR Publications website on July 26, 2023, together with the publication of this correction notice. Because this was made after submission to PubMed, PubMed Central, and other full-text repositories, the corrected article has also been resubmitted to those repositories.

