# Supplementary figures and images for: Effectiveness of Silver Diamine Fluoride for Early Childhood Caries Among Children Aged 24 to 72 Months: Protocol for a Randomized Controlled Trial
Source: JMIR Res Protoc. 2023 Jun 7;12:e46144. doi: 10.2196/46144 (PMC10285618; doi:10.2196/46144)

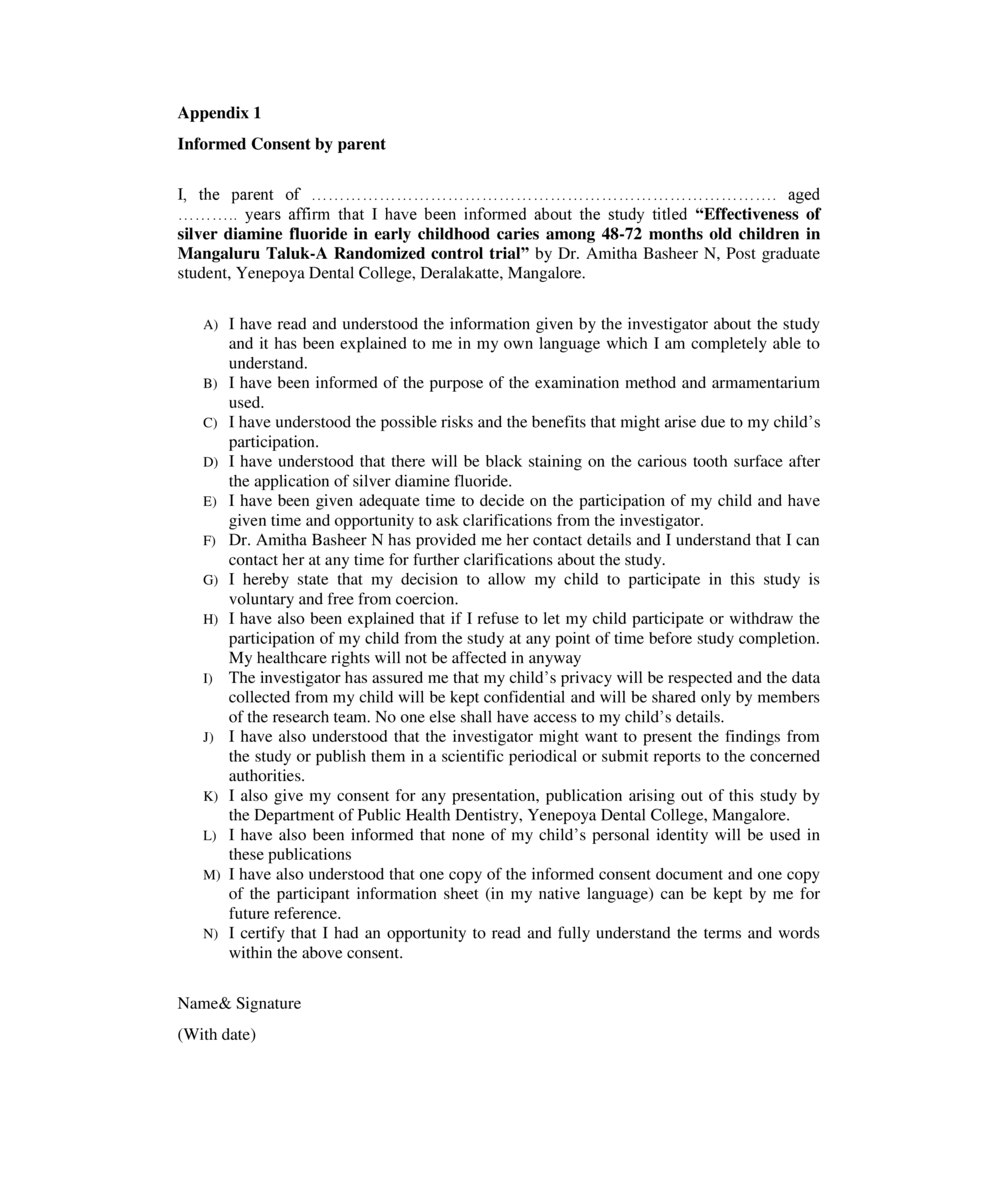

Supplement: Multimedia Appendix 1 [file resprot_v12i1e46144_app1.png]
